# Editorial: Inherited Arrhythmias of the Cardiac Sodium Channel Na_v_1.5

**DOI:** 10.3389/fphys.2021.716553

**Published:** 2021-08-04

**Authors:** Mohamed-Yassine Amarouch, Elena V. Zaklyazminskaya, Jean-Sébastien Rougier

**Affiliations:** ^1^R.N.E Laboratory, Multidisciplinary Faculty of Taza, University Sidi Mohamed Ben Abdellah, Fez, Morocco; ^2^Medical Genetics Laboratory, Petrovsky National Research Centre of Surgery, Moscow, Russia; ^3^Institute of Biochemistry and Molecular Medicine, University of Bern, Bern, Switzerland

**Keywords:** cardiac channelopathies, electrophysiology, inherited arrhythmias, SCN5A, Na_v_1.5

Ion channels are pore-forming transmembrane proteins involved in the transport of ions across cell membranes according to their electrochemical gradients. In cardiomyocytes, the activity of these proteins maintains the resting membrane potential and generates action potentials. Thereby, ion channels play a key role in the excitability of the heart.

Ion channel dysfunction is linked with a broad range of inherited arrhythmias that may lead to sudden cardiac death. These disorders are formerly known as channelopathies and are commonly associated with the presence of mutations in genes encoding cardiac ion channels and/or their interacting proteins (Skinner et al., 2019). As a consequence, functional alterations or mislocalization of ion channels or their regulatory proteins might deeply affect the action potential and promote life-threatening ventricular arrhythmias through changes in intracellular calcium handling (Landstrom et al., 2017).

In the heart, Na_v_1.5 represents the preponderant isoform of the voltage-gated sodium channels. It is responsible for the fast-initial depolarization phase of action potential and as such represents a critical determinant of the excitability and conduction of the electrical impulse through the myocardium. Therefore, Na_v_1.5 dysfunction has been linked with several inherited cardiac arrhythmias. On the one hand, a gain-of-function mutations of the *SCN5A* gene, which encodes the α-subunit of Na_v_1.5, are associated with the congenital long QT syndrome, atrial fibrillation, multifocal ectopic Purkinje-related premature contractions, and exercise-induced polymorphic ventricular tachycardia (Amarouch and Abriel, 2015; Verkerk et al., 2018). On the other hand, loss-of-function mutations of this channel are linked with Brugada Syndrome (BrS), sick sinus syndrome, and cardiac conduction diseases (Verkerk et al., 2018). Moreover, Na_v_1.5 malfunction may also lead to structural heart diseases such as dilated cardiomyopathy and arrhythmogenic right ventricular cardiomyopathy/dysplasia (Verkerk et al., 2018; Jordan et al., 2021).

The purpose of this Frontiers Research Topic on inherited arrhythmias of the cardiac sodium channel Na_v_1.5 is to present recent findings, leading us to have a better understanding of the cardiac sodium channel physiology, and highlighting the pathophysiological mechanisms underlying arrhythmias related to cardiac sodium channelopathies. These investigations can contribute significantly to improving decision-making along the whole patient pathway by targeting the biological effects of the disease-causing mutations.

In this Frontiers Research Topic Dong et al. provide a review on the life cycle of the cardiac voltage-gated sodium channel Na_v_1.5. These authors focused on the different phases of the Na_v_1.5 life cycle and summarized *SCN5A*-related diseases and novel therapeutic strategies targeting Na_v_1.5.

Tse et al. present a territory-wide study from Hong Kong and analyzed the genetic composition of BrS patients, who underwent genetic testing over a 21-year period. For this purpose, a total of 65 patients were included and analyzed retrospectively. This study identifies six novel variants in the *SCN5A* gene, which have not been reported in cohorts outside of the Hong Kong region.

Regarding original research articles, Hichri et al. developed a novel computational approach to address an important topic related to a recent finding in the field of sodium channel biophysics. Clatot et al. (2017) report that sodium channels, including Na_v_1.5, act as a dimer. Their α-subunits interact physically with each other via the cytoplasmic protein 14-3-3, leading to coupled channel gating. These findings strongly suggest that sodium channels operate and gate as dimers rather than non-interacting entities. In this context, Hichri et al. developed a novel approach, integrating the notion of sodium channel dimer as the functional unit of the sodium current. The authors investigated whether the newly identified channel-channel interactions can contribute to the negative dominant effect of cardiac sodium channel variants such as Na_v_1.5-p.L325R. The simulation results suggest that interactions with the variant channel may contribute to the negative-dominant effect.

In the same context, Zheng et al. investigate the dominant negative effect mechanisms produced by the *SCN5A* splice variant E28D. This variant results in the truncated sodium channel Na_v_1.5-p.G1642X, which is significantly upregulated in human Heart Failure (HF). These authors demonstrate that the Na_v_1.5-p.G1642X channel interacts with the WT-Na_v_1.5 and thereby exerts a dominant-negative effect, contributing to the decrease in I_Na_ seen in HF. Furthermore, Zheng et al. show that the sodium channel polymorphism p.H558R can rescue the loss-of-function related to the Na_v_1.5-p.G1642X dominant-negative effect by impairing the biophysical coupling between the splice variant and the WT channel.

Also related to the same topic, Doisne et al. investigate the dominant-negative effect of the BrS Na_v_1.5-p.R104W variant in a murine model using adeno-associated viruses. The cardiac sodium current of p.R104W injected mice was significantly decreased. Moreover, the overexpression of this variant in normal hearts led to a decreased expression of Na_v_1.5. Altogether, these results demonstrated an *in*-*vivo* dominant-negative effect of Na_v_1.5-p.R104W channels on the endogenous ones.

Finally, Ghovanloo et al. characterize a novel *SCN5A* variant, p.T1857I, identified in a family with a CPVT-like phenotype, and located in the C-terminus of Na_v_1.5. The functional characterization of Na_v_1.5-p.T1857I revealed significant positive shifts in voltage-dependences of both activation and inactivation. Moreover, a delayed recovery from fast inactivation was observed in the mutated condition, while action potential simulations suggest the occurrence of ventricular after depolarization, predisposing carriers to cardiac arrhythmias.

## Conclusion

This Research Topic underscores the pathophysiological implications of rare *SCN5A* variants in cardiac arrhythmia. Many of the published studies highlight the molecular complexity underlying the effect of some rare *SCN5A* variants, especially the contribution of the newly identified α-α subunit interactions to Na_v_1.5 gating and the negative dominant effect.

## Author Contributions

M-YA, EZ, and J-SR have made a direct contribution to the work and approved it for publication. All authors contributed to the article and approved the submitted version.

## Conflict of Interest

The authors declare that the research was conducted in the absence of any commercial or financial relationships that could be construed as a potential conflict of interest.

## Publisher's Note

All claims expressed in this article are solely those of the authors and do not necessarily represent those of their affiliated organizations, or those of the publisher, the editors and the reviewers. Any product that may be evaluated in this article, or claim that may be made by its manufacturer, is not guaranteed or endorsed by the publisher.
